# Intrinsic disorder in putative protein sequences

**DOI:** 10.1186/1477-5956-10-S1-S19

**Published:** 2012-06-21

**Authors:** Uros Midic, Zoran Obradovic

**Affiliations:** 1 Fels Institute for Cancer Research & Molecular Biology, Temple University School of Medicine, 3307 N. Broad St, Philadelphia, PA 19140, USA; 2 Center for Data Analytics and Biomedical Informatics, Temple University, Room 303 Wachman Hall, 1805 N. Broad St, Philadelphia, PA 19122, USA

## Abstract

**Background:**

Intrinsically disordered proteins (IDPs) and regions (IDRs) perform a variety of crucial biological functions despite lacking stable tertiary structure under physiological conditions in vitro. State-of-the-art sequence-based predictors of intrinsic disorder are achieving per-residue accuracies over 80%. In a genome-wide study of intrinsic disorder in human genome we observed a big difference in predicted disorder content between confirmed and putative human proteins. We investigated a hypothesis that this discrepancy is not correct, and that it is due to incorrectly annotated parts of the putative protein sequences that exhibit some similarities to confirmed IDRs, which lead to high predicted disorder content.

**Methods:**

To test this hypothesis we trained a predictor to discriminate sequences of real proteins from synthetic sequences that mimic errors of gene finding algorithms. We developed a procedure to create synthetic peptide sequences by translation of non-coding regions of genomic sequences and translation of coding regions with incorrect codon alignment.

**Results:**

Application of the developed predictor to putative human protein sequences showed that they contain a substantial fraction of incorrectly assigned regions. These regions are predicted to have higher levels of disorder content than correctly assigned regions. This partially, albeit not completely, explains the observed discrepancy in predicted disorder content between confirmed and putative human proteins.

**Conclusions:**

Our findings provide the first evidence that current practice of predicting disorder content in putative sequences should be reconsidered, as such estimates may be biased.

## Background

Intrinsically disordered proteins (IDPs) are proteins that lack stable tertiary structure under physiological conditions in vitro [1]. They are also known by other names, including natively denatured [2], natively unfolded [3], intrinsically unstructured [4], and natively disordered [5]. IDPs can be wholly disordered or partially disordered, where we can identify intrinsically disordered regions (IDRs) and ordered regions. Although they lack stable tertiary structure, the functional repertoire of IDPs complements the functions of ordered proteins. IDPs are involved in a number of crucial biological functions including regulation, recognition, signaling and control.

**Figure 1:**
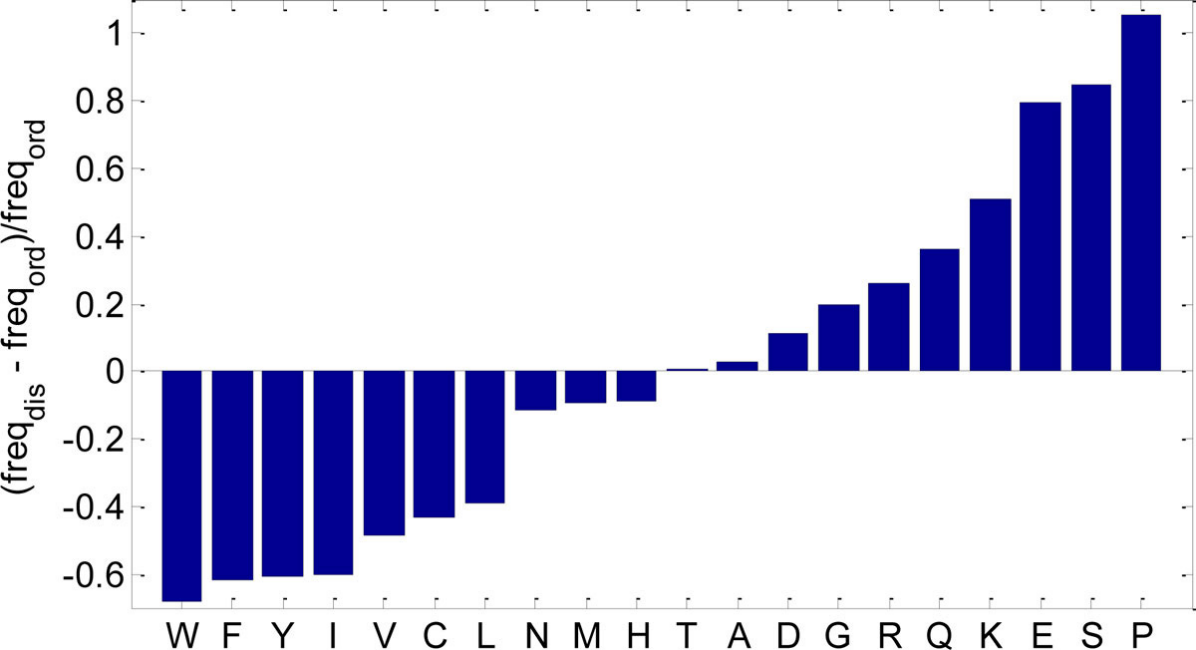
**Comparison of amino-acid frequencies in predicted disordered (*freq_dis_*) and ordered (*freq_ord_*) regions of human proteins**. Amino acids are sorted by the relative difference (*freq_dis _*-*freq_ord_*)/*freq_ord_*.

There are several crucial differences between amino acid sequences of IDPs/IDRs and structured globular proteins and domains. These differences include divergence in amino acid composition and sequence complexity, and consequently in physicochemical properties like hydrophobicity, aromaticity, charge, and flexibility index value [6]. For example, IDPs possess a low content of N and of the cross-linking C residues and are significantly depleted in bulky hydrophobic (I, L, and V) and aromatic amino acid residues (W, Y, and F), which form and stabilize the hydrophobic cores of folded globular proteins. These amino acids have been called *order-promoting amino acids*. On the other hand, IDPs/IDRs are substantially enriched in polar and charged amino acids (R, Q, S, E, and K) and in structure-breaking G and P residues, collectively called *disorder-promoting amino acids *[1,7,8]. The difference in amino acid composition between predicted ordered and predicted disordered regions in human proteins is illustrated in Figure 1.

Thus, in addition to the well-known "protein folding code" stating that all the information necessary for a given protein to fold is encoded in its amino acid sequence [9], "protein non-folding code" has been proposed, according to which the propensity of a protein to stay intrinsically disordered is likewise encoded in its amino acid sequence [10,11]. This has been utilized to develop numerous predictors of intrinsic disorder (ID), which achieve over 80% of per-residue accuracy [12].

**Figure 2:**
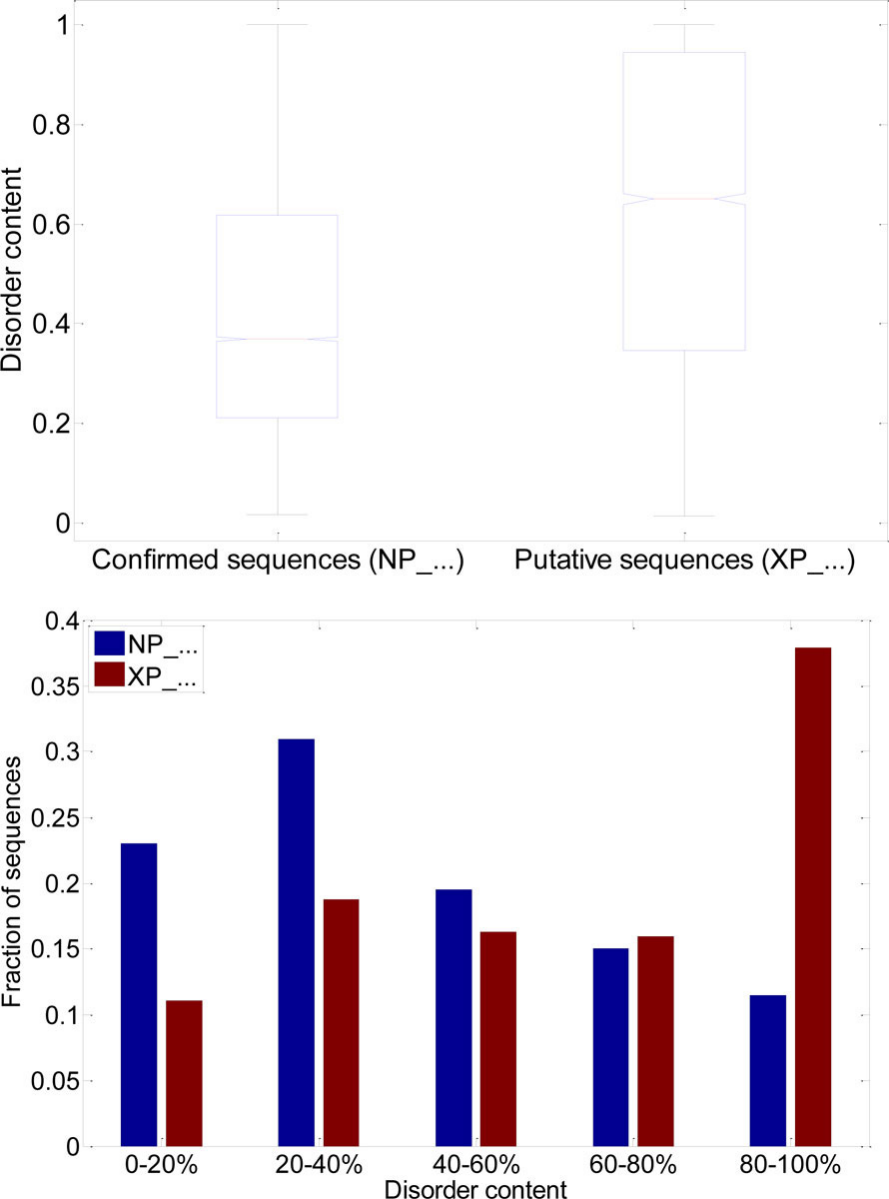
**Comparison of predicted disorder content distributions in the confirmed human protein sequences (NP_...) and the putative human protein sequences (XP_...)**. Protein sequences were taken from [16]. Top: Boxplot comparison of distributions of disorder content. Bottom: Comparison of histograms with respect to the disorder content.

Large-scale genome-wide prediction of ID has been used previously to confirm the ubiquity of ID [13], and to compare the abundance of ID in various genomes and groups of genomes [14,15]. In a previous study [16] we applied a per-residue predictor of ID to human proteins available in NCBI database, to compare disorder content in various classes of human proteins. One intriguing result was the vast discrepancy in predicted disorder content between the protein sequences with IDs starting with NP (protein records in advanced stage of the curation process, further referred as *NP sequences *and *NP class*) and protein sequences with IDs starting with XP (protein records in early stages of the curation process, further referred as *XP sequences *and *XP class*). The difference in distributions of predicted disorder content for NP class and XP class of sequences is shown in Figure 2; we define *disorder content *(DC) as the fraction of residues in a sequence that are predicted to be disordered. Note that XP class has a low fraction of proteins predicted as fully and mostly ordered (DC < 20%), and that there is more than two-fold difference in fractions of proteins predicted as fully and mostly disordered (DC > 80%) in XP class compared to NP class.

**Figure 3:**
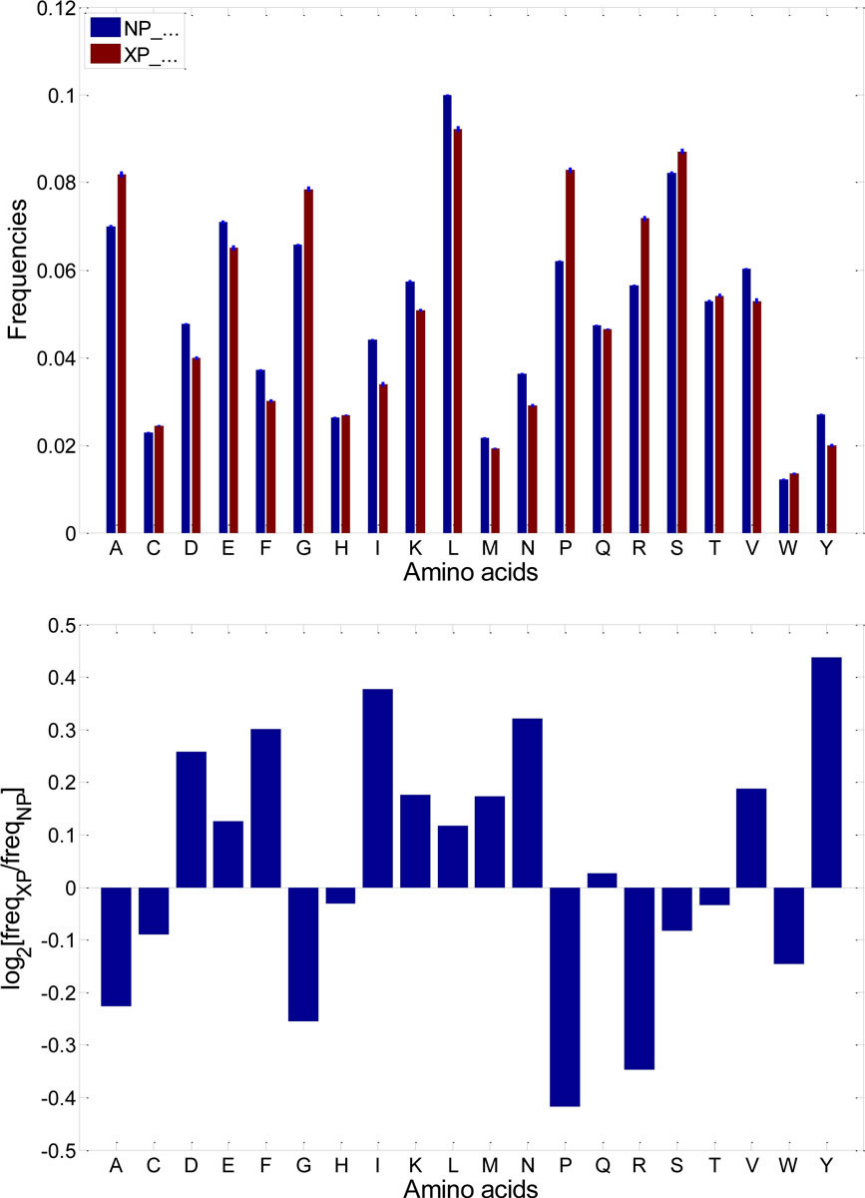
**Comparison of amino acid compositions in the confirmed human protein sequences (NP_...) and the putative human protein sequences (XP_...)**. Protein sequences were taken from [16]. Top: Direct comparison of frequencies (error bars are too small to be visible). Bottom: Log_2_-ratios of frequencies; amino acids with positive values are enriched in confirmed sequences, amino acids with positive values are enriched in putative sequences.

The simplest explanation for this is that the automated annotation procedure has a high error rate that introduces a large number of incorrect amino acid sequences. Alternatively, this dramatic difference in the level of predicted ID between the experimentally and automatically identified proteins could be due to the bias of the existing identification techniques toward the ordered proteins. To some extent this resembles a problem the Structural Genomics Initiative Centers are facing, where the use of the traditional target search criteria (mostly based on the sequence identity) and protein purification and isolation methods generated mostly ordered targets, whereas alternatively identified and purified proteins awaiting structure determination were richer in disorder than an average protein in PDB [17,18]. It has been pointed out that this bottleneck was determined by the strategy chosen where in efforts to identify proteins with novel folds researchers started with proteins having amino acid sequences unlike those of proteins with known 3D structures [17,18]. In a similar manner, traditional experimental approaches developed for protein identification could be biased toward order (as ordered well-folded proteins were at the research focus for many years), whereas predictive tools are mostly dealing with the remaining part of the proteomes and therefore are inevitably identifying more disordered proteins. Two groups of sequences also have significantly different amino acid distributions (Figure 3). The enrichment of several amino acids in confirmed sequences (F, I, L, N, V, Y) and in putative sequences (G, P, R, S) is consistent with the order promoting vs. disorder promoting classification of amino acids.

The predicted sequences were unevenly distributed between disease-related and disease-unrelated proteins. In fact, the majority of the putative sequences were products of the non-disease genes. Therefore, including such sequences into the data set would introduce significant bias for disorder in the non-disease gene part of the data set. Based on these observations, we decided to exclude such sequences from the final datasets.

Gene finding is the problem of predicting the positions of genes, and the positions of exons and introns inside the genes, for a given genomic sequence. Most predictors use Bayesian networks, such as Interpolated Markov Models [19], Generalized Hidden Markov Models [20], and Generalized Pair HMMs [21]. These predictors exploit the following findings: 1) many signals involved in gene expression (e.g. promoters, splice junctions) exert specific patterns, known as motifs, and can be predicted from sequence, 2) protein-coding DNA have statistical properties (such as amino acid composition, length) that distinguish them from non-coding DNA, 3) signals and statistical properties are often conserved across related sequences (intra- and inter-species). From the domain experts' point of view, these prediction models perform well, as they provide important guidelines for experimental research, where predicted putative sequences are confirmed or refined. However, inclusion of these putative sequences in large-scale analysis of ID is questionable. Even when predicted exons of a predicted protein sequence overlap with true exons, the overlap can be partial and non-coding DNA may be included in the predicted exons. Another possibility is that in predicted protein sequence, true exons are translated in wrong reading frame. Therefore, predicted protein sequences contain regions that come from non-coding genomic regions or incorrectly translated coding regions, and are not present in true protein sequences. In further text we will refer to them as nonsense regions/sequences. Nonsense regions do not exist in real proteins, and the hypothetical structure they would conform to if they were synthesized is uncertain. Therefore, any prediction of structure - including prediction of intrinsic disorder - for nonsense regions and sequences is not valid. Inclusion of such sequences in genome-wide analysis of intrinsic disorder can possibly substantially bias the estimate of ID content in a genome. In [16] we decided to exclude XP sequences from analysis of ID in human genome. On the other hand, their exclusion from genome-wide analysis can also give an unrealistic estimate of ID content, especially if the proportion of incorrectly annotated unconfirmed sequences is high. If the higher predicted disorder content in XP sequences is realistic, then their exclusion can negatively bias the estimate of ID content in the genome.

In this paper we explore the relationship between nonsense regions in XP sequences - introduced through errors made by gene finding procedures - and intrinsic disorder. In addition to the difference in amino acid composition between NP and XP sequences (further elaborated in the Results section), we assumed that nonsense regions follow a different amino acid composition than true protein sequences. Therefore, instead of testing and improving the gene finding algorithms, we investigate whether nonsense regions can be detected from amino acid sequence, similarly to prediction of intrinsic disorder.

We developed a two-class predictor that aims at distinguishing true protein sequences from nonsense regions in putative sequences. Since no data is easily available about which regions of XP sequences are nonsense, we constructed synthetic nonsense sequences from mRNAs of the true protein sequences that form the other class.

The methodology that was used to create the synthetic nonsense sequences, train and evaluate the nonsense predictor, and analyze results of the predictor for XP sequences is described in the Methods section. Results section presents more details on the comparison of amino acid sequence composition, results of predictor evaluation, comparison of nonsense prediction in different classes of sequences, and the analysis of relationship between nonsense prediction and disorder prediction. This is followed by brief Discussion and Conclusions sections.

This paper is a substantial extension of the preceding conference paper [22]. The dataset that was used in the initial attempt at performing this analysis was based on the dataset used in [16], which was retrieved from the NCBI database in 2007, and included only the human genome. Since additional information about genes and proteins was required to answer open questions and improve several shortcomings of the setup for the initial study, we downloaded all the necessary information from the NCBI database again in 2011 and performed analysis with improved methodology and, in addition to the updated human dataset, also three new datasets: mouse, fruitfly and zebrafish. This paper presents the methodology and the results of the extended study. However, the old methodology and results are also mentioned where appropriate, since the comparison of the results gives an important insight into the trends of the development of the NCBI databases that are relevant for the topic of this paper.

## Methods

### Dataset and creation of synthetic nonsense sequences

We created four datasets, one for each of the following species: Homo sapiens (human), Mus musculus (mouse), Drosophila melanogaster (fruitfly), Danio rerio (zebrafish). For each of the organisms, we downloaded genomic records with sequences and annotation about all genes with RefSeq protein records from the NCBI database. These records contain the genes' nucleotide sequences, as well as position of all parts of mRNA sequences: 3' and 5' UTRs (untranslated regions) and coding regions (exons). From this information we could also easily identify intronic regions. For the control/negative class of true proteins we selected either NP protein sequences that are listed as single isoforms of respective genes (i.e. the genes are not known to be involved in alternative splicing), or representative sequences compiled from multiple NP sequences for genes with multiple isoforms (i.e. alternatively spliced); a representative sequence was compiled by translating all exon regions in a genes sequence. The only exceptions were the alternatively spliced genes for which at least one of the exons was translated in a different codon alignment in different isoforms; such genes were not used in this study.

**Figure 4:**
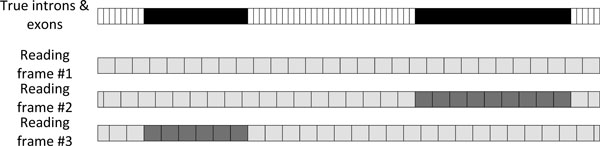
**Illustration of the procedure to synthesize nonsense protein sequence from genomic sequences with confirmed exon positions**. A genomic sequence with known exon positions (black, dark grey) is read and translated in three different ways, with different starting codon positions. Parts of an obtained amino acid sequence that align perfectly to parts of the confirmed protein are discarded (dark grey), while the remainder is kept as a synthesized nonsense sequence (light grey). The schema is simplified, true sequences and exons are longer than the ones depicted here.

Nonsense protein sequences for the positive class were synthesized from coding and noncoding regions of the genomic sequences of genes whose representatives form the negative class. The exact locations of exons in these genomic sequences are known, and the exons can only be translated correctly if they are read in one of the three possible reading frames. For a given annotated genomic sequence and the protein it is translated to (top sequence in Figure 4, where exons are shown in black), the procedure to synthesize nonsense sequences was the following:

Crop the gene's nucleotide sequence by removing all nucleotides from noncoding regions that are further than 120 nucleotides away from the closest exon. The obtained nucleotide sequence can be translated in three different reading frames, for each of these three reading frames: 1) Translate the codons into amino acids, ignore/discard any stop codons (this amino acid sequence is further referred to as the *candidate sequence*). 2) Align the candidate sequence to the true protein sequence. 3) Identify any parts of the candidate sequence that are perfectly matched to the true protein sequence, and are at least 10 amino acids long (shown as dark gray in Figure 4). These come from true exons that are correctly translated and are therefore removed from the candidate sequence. 4) The remaining parts of the candidate sequence (light gray in Figure 4) are either coming from non-coding regions or from incorrectly translated exons; therefore they can be considered to be nonsense sequences.

This procedure produces three nonsense sequences for each true protein sequence. As an additional set, we selected representative sequences for genes with XP protein sequences the same way as for genes with NP protein sequences. We discarded short sequences for which construction of input features for prediction is not viable. The overview of the number of sequences in the three groups (NP, XP, synthetic nonsense) for four genomes is given in Table 1.

**Table 1:** Overview of numbers of sequences in datasets for nonsense prediction

Organism	NP sequences	XP sequences	Synthetic nonsense sequences
Homo sapiens	14353	307	42923
Mus musculus	14661	799	43808
Drosophila melanogaster	12190	0	36240
Danio rerio	9331	7867	27897

Sequences from both parts of the dataset and the additional set were preprocessed to construct predictive features, similarly to how features are constructed for PONDR family of ID predictor [7,12,23,24]. For each fixed residue, a window of size 41 was positioned centered at the fixed residue. Amino acids in the window were counted and their frequencies were calculated; this produced 20 features that correspond to amino acid composition. Entropy was calculated from 20 amino acid frequencies; this feature measures local complexity of amino acid sequence. Local flexibility was approximated as the scalar product of 20 amino acid frequencies and 20 flexibility parameters, which were estimated empirically. Net charge and average hydrophobicity were calculated similarly to flexibility, and their ratio is used as an additional feature. Predictions of ID were obtained with the VSL2B predictor [12]; these predictions are mapped to binary classification by applying the .5 threshold. To summarize the predicted ID in a protein sequence, we used *disorder content *(DC), which is defined as the fraction of residues that are predicted to be in disordered regions. We labeled amino acids in synthetic nonsense sequences with information about their origin, i.e. whether the central nucleotide of the corresponding codon was a part of coding region or non-coding region. For amino acids in all sequences we calculated the distance of the codon from the nearest border between exon and a non-coding region. Both of these labels were later used in balancing of the training set.

The main difference in the above described datasets and the dataset in the initial study [22] is that in the initial study only the mRNA sequences of the human proteins were used as the source for synthesis of nonsense sequences. The translation of non-coding regions was therefore limited only to upstream and downstream untranslated regions (3'UTR and 5'UTR) if they were included in the mRNA sequence at all. We also excluded all genes that were known to be alternatively spliced. The dataset contained 15,124 NP sequences and 45,038 synthetic nonsense sequences, as well as the additional set of 5,243 XP sequences.

### Prediction of nonsense regions in protein sequences

This prediction problem is novel, and therefore we could not utilize any of the existing protein-related prediction tools. Furthermore, we could not compare our results to any previously published results. Our goal was not to develop an optimal predictor, but rather to construct a simple predictor with reasonable accuracy and good balance between sensitivity and specificity. We briefly tested logistic regression and neural networks as the predictive model, with various sets of parameters. Here we present only the parameters that led to the best results that we have obtained. We used neural networks with 20 hidden nodes in a single hidden layer. We always trained ensembles of 10 neural networks, with randomly sampled training and validation sets. The training and validation sets (8% and 2% of the available data respectively) were sampled from the dataset; only 10% of the available data was used per iteration to speed up the training and evaluation process. Because windows used to construct features for neighboring amino-acids were overlapping, the obtained features were similar, and therefore the redundancy allowed for subsampling without significant loss of accuracy.

Both training and validation sets were balanced (i.e. contained equal number of residues from positive and negative class), and samples from both classes were balanced in terms of disorder to include equal number of residues predicted to be ordered and disordered. We further balanced the nonsense class by sampling equal number of residues obtained by translating non-coding regions and residues obtained by translating coding regions. We also balanced both nonsense and true protein class by sampling equal numbers of residues obtained from regions in vicinity of an exon/non-coding region border (50nt or less) and of residues obtained from regions far from such borders (more than 50nt). Targets for residues from two classes were encoded as .1 and .9. In the evaluation phase, the residues were classified by comparing their real-valued predictions with the .5 threshold.

In the initial study [22] we balanced the training dataset only with respect to the class and the predicted disorder, but not with respect to the origin of the amino acids.

### Evaluation

We performed both per-residue and per-sequence evaluation. In per-residue evaluation residues are observed separately, while in per-sequence evaluation predictions for all residues in a sequence are aggregated into one prediction (mean of per-residue predictions) and compared to a threshold. We used 10-fold cross-validation to evaluate the predictor, and the dataset was partitioned into 10 subsets so that residues from the same sequence were always members of the same subset. This partitioning both enables per-protein prediction and ensures fair testing in per-residue prediction, since neighboring residues in a sequence have similar input features and in most cases equal target values, and should therefore always be in the same subset. We used two indicators of nonsense prediction level in a sequence. We define *nonsense content *as the fraction of predicted nonsense residues in a sequence; this indicator is analogous to disorder content. Another indicator is the mean of (real-valued) per-residue nonsense predictions in the sequence. Both indicators were used to compare results of prediction for NP and XP sequences.

To analyze the impact of input features for prediction of nonsense, we used approximation of partial derivatives of prediction function. Partial derivative of prediction function *pred *with respect to *i*-th feature *f_i _*at point *x *was approximated as $\partial pred_{f_{i}}\left(x\right)\approx \left(pred\left(x+\varepsilon_{i}\right)-pred\left(x\right)\right)/\varepsilon$, where *ε_i _*= *ε*(0, ...,1, ...,0) is the vector with value *ε *at *i*-th elementh and value 0 at all other elements. The mean of such estimates for feature *f_i _*over all points in the dataset $\sum_{j=1}^{n}\partial pred_{f_{i}}\left(x_{j}\right)/n$ was then used to estimate both the impact (absolute value) and the direction (sign) of contribution of feature *f_i _*to prediction function.

## Results

**Figure 5:**
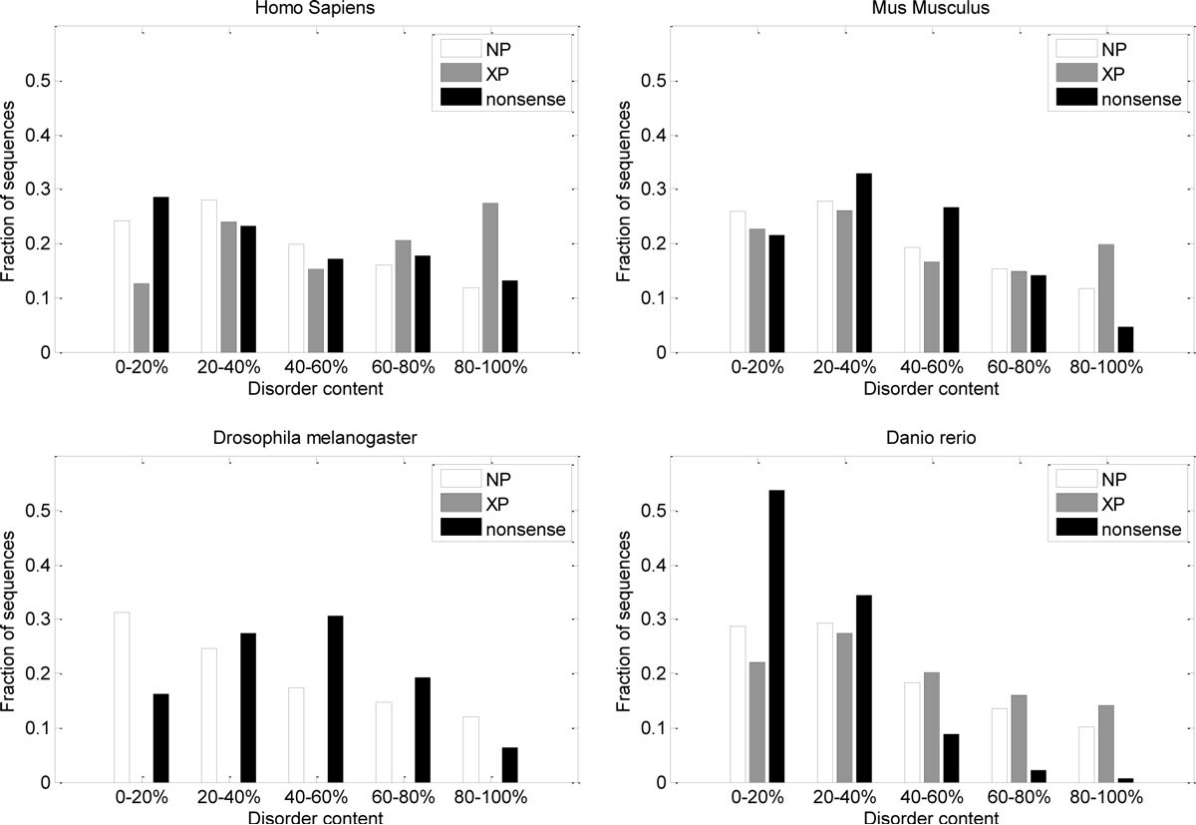
**Distributions of predicted disorder content (DC) in confirmed proteins (NP), putative proteins (XP), and synthetic nonsense sequences**. Histograms show fractions of sequences with various levels of disorder content.

The motivation for this study was the discrepancy in predicted disorder content between NP and XP sequences. The same difference is preserved in the dataset for Homo sapiens (Figure 5), although there is a change in the distribution of disorder content for XP sequences. There are also differences in distributions of disorder content between NP and XP sequences for Mus musculus and Danio rerio (Figure 5), but they are not as large as for Homo sapiens. The distribution curve for disorder content in synthetic nonsense sequences in Danio rerio is strongly skewed.

**Figure 6:**
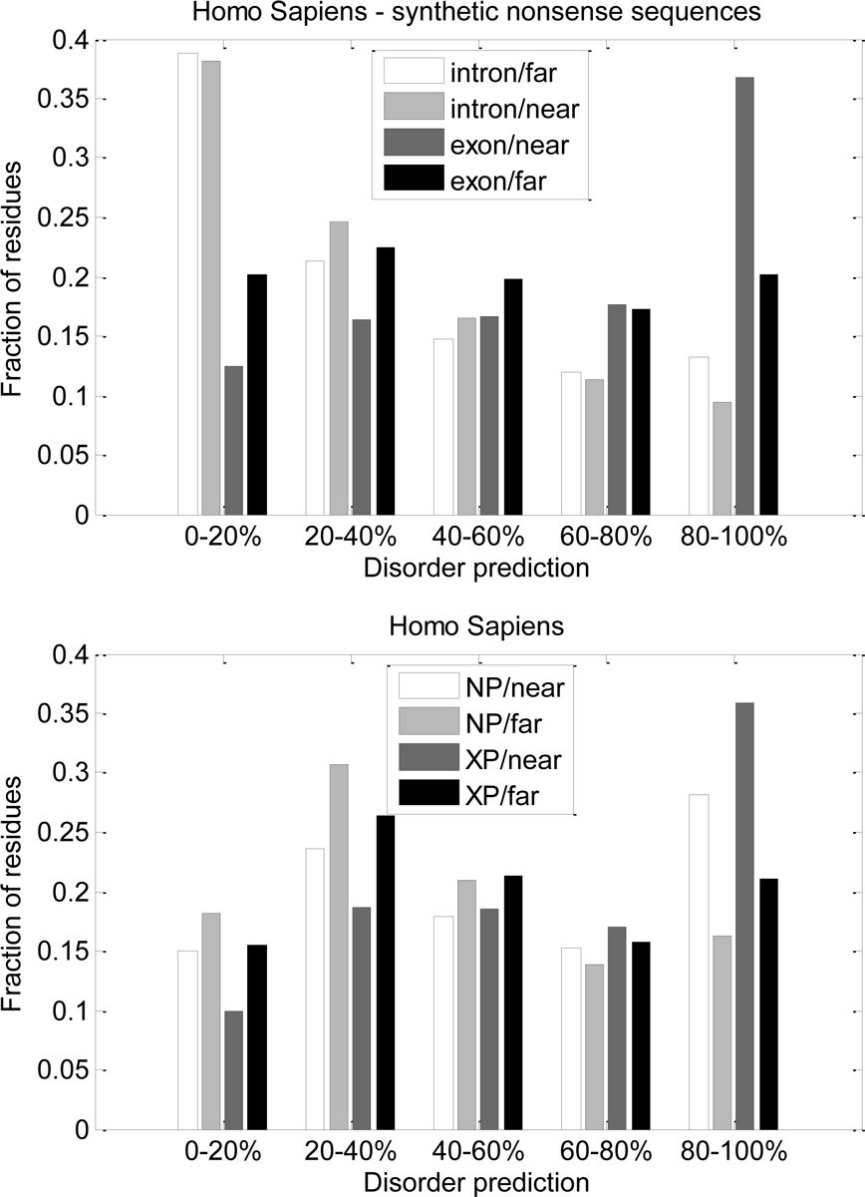
**Distributions of predicted disorder in human synthetic nonsense, NP and XP sequences - comparison by position of codons in genomic sequences**. Residues were grouped by the positions of their codons in genomic sequences (non-coding or exons, near or far from non-coding/exon border). Histograms show fractions of residues with various levels of disorder prediction.

In the synthetic nonsense sequences for Homo sapiens, we can observe a large difference in distributions for residues originating from non-coding regions and exons (Figure 6, top). Distribution for the residues originating from non-coding regions is strongly biased towards order. Distribution of disorder content for residues originating from coding regions further from exons' borders (i.e. in the middle of exons) is fairly uniform. Unlike for the residues originating from non-coding regions, distribution of disorder content for residues originating from coding regions near the exons' borders is strongly biased towards disorder. This is also preserved for NP and XP sequences (Figure 6, bottom), although XP sequences have higher fraction of residues with high levels of disorder prediction. These differences in distributions of disorder content were the reason for the additional balancing of the dataset introduced after the initial study [22].

### Evaluation of nonsense sequence predictor

The results of 10-fold cross-validation evaluation of nonsense predictors are summarized in Table 2. Since the positive class is much larger than the negative class, we measured specificity (true negative rate, accuracy on the negative class) and sensitivity (true positive rate, accuracy on the positive class) separately and used the average value of sensitivity and specificity as the adjusted measure of accuracy. We also report area under ROC curve (AUC). For per-residue prediction we also perform separate evaluation of predictors for disordered and ordered regions, separate evaluation for nonsense regions originating from exons and nonsense regions originating from non-coding regions (in both cases the negative class remains the same, i.e. contains all NP sequences).

**Table 2:** 10-fold cross-validation evaluation of per-residue and per-protein nonsense sequence predictors

	Specificity	**Sensitivity**.	Accuracy = (spec+sens)/2	Area under curve
**Homo sapiens**				

**Per-residue**				
**Overall**	**83.47%**	**84.42%**	**83.94%**	**0.9189**
Ordered regions	82.35%	84.03%	83.19%	0.9119
Disordered regions	84.82%	85.04%	84.93%	0.9276
Nonsense ~ introns	83.47%	84.15%	83.81%	0.9174
Nonsense ~ exons	83.47%	84.94%	84.20%	0.9217

**Per-protein**	**94.43%**	**98.81%**	**96.62%**	**0.9933**

**Mus musculus**				

**Per-residue**				
**Overall**	**82.28%**	**83.28%**	**82.78%**	**0.9087**
Ordered regions	81.15%	82.94%	82.05%	0.9016
Disordered regions	83.69%	83.88%	83.79%	0.9182
Nonsense ~ introns	82.28%	81.92%	82.10%	0.9022
Nonsense ~ exons	82.28%	85.91%	84.09%	0.9213

**Per-protein**	**94.02%**	**98.85%**	**96.43%**	**0.9938**

**Drosophila melanogaster**				

**Per-residue**				
**Overall**	**84.57%**	**87.14%**	**85.86%**	**0.9360**
Ordered regions	82.05%	85.59%	83.82%	0.9187
Disordered regions	87.70%	88.86%	88.28%	0.9538
Nonsense ~ introns	84.57%	81.18%	82.88%	0.9105
Nonsense ~ exons	84.57%	90.04%	87.31%	0.9485

**Per-protein**	**96.97%**	**97.54%**	**97.26%**	**0.9938**

**Danio rerio**				

**Per-residue**				
**Overall**	**83.29%**	**87.12%**	**85.20%**	**0.9297**
Ordered regions	80.80%	88.41%	84.61%	0.9262
Disordered regions	86.85%	82.38%	84.61%	0.9266
Nonsense ~ introns	83.29%	88.00%	85.64%	0.9338
Nonsense ~ exons	83.29%	85.47%	84.38%	0.9220

**Per-protein**	**95.98%**	**99.60%**	**97.79%**	**0.9980**

All indicators of predictor's performance on Homo sapiens dataset showed a small improvement compared to the results of the initial study [22]. To test the reason behind that improvement we trained a predictor on the Homo sapiens dataset for which the training dataset was only balanced with respect to true/nonsense and order/disorder criteria, but not with respect to the non-coding/exonic origin and the near/far from non-coding-exon border criteria. The evaluation of these predictors (results not shown) showed similar improvement compared to results for the initial study [22]. Therefore we can conclude that additional balancing did not directly affect the performance of the predictor. Instead, the improvement in performance can be attributed to one of the following: 1) changes in the NCBI dataset that occurred over last three years (refinement of NP sequences and upgrading of XP sequences to NP status), 2) inclusion of more intronic regions into the synthetic nonsense part of the dataset, 3) inclusion of sequences with alternative splicing.

### Comparison of predicted nonsense in NP and XP sequences

As a part of the 10-fold cross-validation process, we obtained predictions for all NP and synthetic nonsense sequences. We could then use all 10 predictors as an ensemble for prediction on XP sequences, since they were not used in training; the ensemble predictor is expected to perform at least as well as its component predictors [25].

**Figure 7:**
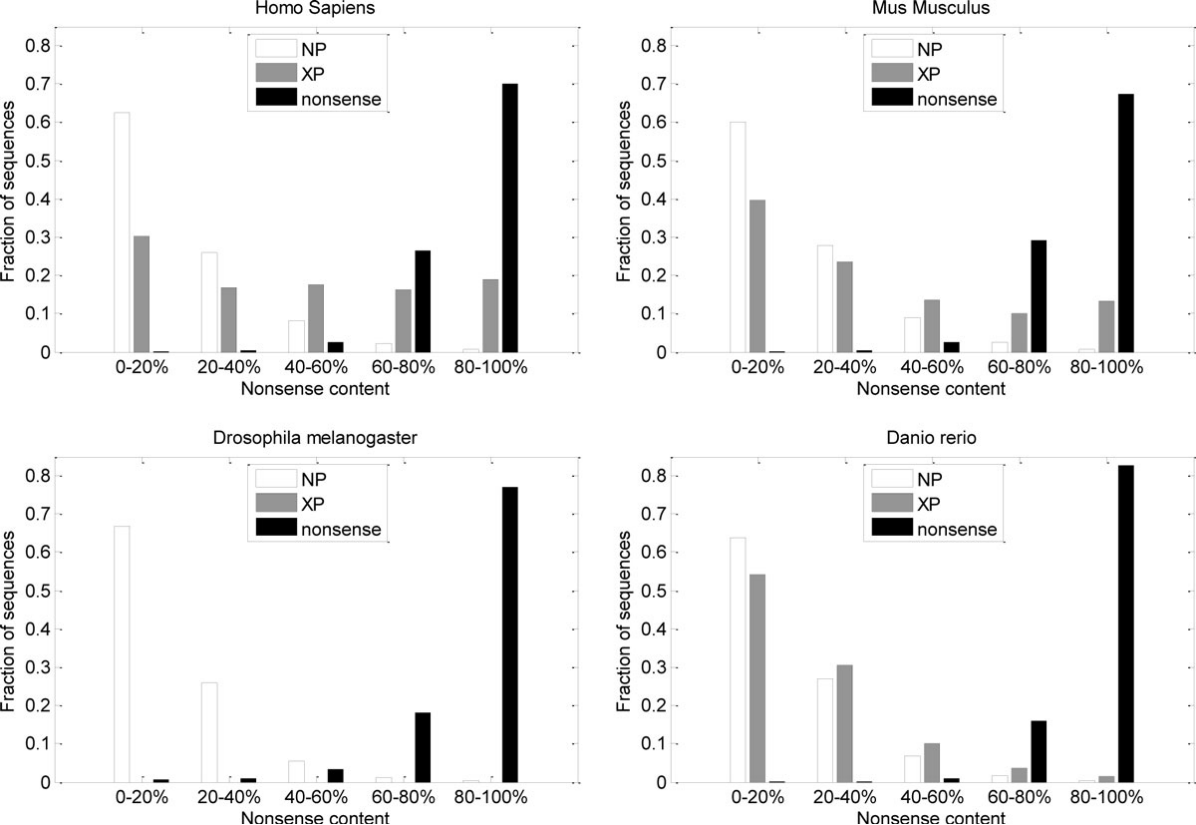
**Distributions of predicted nonsense content (NC) in confirmed proteins (NP), putative proteins (XP), and synthetic nonsense sequences**. Histograms show fractions of sequences with various levels of nonsense content.

We calculated nonsense content for all NP, XP and synthetic nonsense sequences. The distributions of nonsense content in the three groups of sequences (NP, XP, synthetic nonsense) for four datasets are compared in Figure 7. Difference between NP and synthetic nonsense sequences is expected in accordance with predictor evaluation results. However, the significant increase in nonsense content for human XP sequences, compared to NP sequences, cannot be explained by the design of the predictor or attributed to noise. With respect to the input features, derived from amino acid sequence, significant portion of human XP sequence regions are more similar to synthetic noise sequences than to NP sequences. There is also a (much smaller) difference in nonsense content between mouse NP and XP sequences. However, distributions of nonsense content for NP and XP sequences in Danio rerio are almost the same.

Comparison between nonsense content prediction for NP and XP sequences and the effects of the choice of threshold is further elaborated in Table 3, which lists fractions of sequences that are predicted to be "mostly nonsense" (i.e. nonsense content is greater than some threshold). Here we can again observe the drop of the fraction for XP sequences in Mus musculus and especially in Danio rerio.

**Table 3:** Comparison of fractions of NP, XP and synthetic nonsense sequences with nonsense content greater than threshold

Organism	Threshold	NP	XP	**Synth. nons**.
Homo sapiens	0.4	11.34%	52.77%	99.38%
**Homo sapiens**	**0.5**	**5.98%**	**44.30%**	**98.64%**
Homo sapiens	0.6	3.07%	35.18%	96.71%

Mus musculus	0.4	12.22%	36.92%	99.37%
**Mus musculus**	**0.5**	**6.55%**	**29.41%**	**98.70%**
Mus musculus	0.6	3.20%	23.28%	96.77%

Drosophila melanogaster	0.4	7.37%		98.48%
**Drosophila melanogaster**	**0.5**	**3.71%**		**97.40%**
Drosophila melanogaster	0.6	1.72%		95.12%

Danio rerio	0.4	8.95%	15.32%	99.78%
**Danio rerio**	**0.5**	**4.39%**	**9.01%**	**99.53%**
Danio rerio	0.6	2.10%	5.17%	98.76%

We also compared the total fractions of residues predicted to be in nonsense regions (Table 4). While the margin between total nonsense content between NP and synthetic nonsense sequences, which equals (*sensitivity*+*specificity*)-1, is increased for Homo sapiens compared to the dataset from the initial study [22], the margin between total nonsense content in XP and NP sequences is decreased (from 20.05% to 18.09%). The same margin is further decreased for Mus musculus, and almost non-existent for Danio rerio.

**Table 4:** Total (per-residue) predicted nonsense content in NP, XP and nons sequences, and the margin of nonsense content between NP and XP, and between NP and synthetic nonsense sequences

Organism	NC_NP	NC_XP	NC_XP - NC_NP	NC_nons	NC_nons - NC_NP
Homo sapiens	16.57%	34.65%	18.09%	84.42%	67.86%
Mus musculus	17.75%	27.21%	9.46%	83.29%	65.54%
Drosophila melanogaster	15.41%			87.15%	71.74%
Danio rerio	16.69%	18.81%	2.12%	87.13%	70.44%

### Relationship between prediction of nonsense in XP sequences and prediction of intrinsic disorder

After the computational experiments have indicated that human (and to some extent mouse) XP sequences contain substantial fraction of nonsense regions, the important question is how these regions affect the prediction of disorder content in XP sequences. In human XP sequences, 55.53% of all residues are predicted to be disordered. In regions of human XP sequences that are predicted to be nonsense the fraction of predicted ID residues is increased to 64.87%, while in regions predicted not to be nonsense, the fraction of predicted ID residues is only 50.58%. It is interesting to note here that in the mouse dataset, predicted fraction of ID residues is very similar in predicted nonsense regions of XP sequences (48.79%), regions of XP sequences that are predicted not to be nonsense (49.09%) and overall XP sequences (49.01%). Furthermore, in the zebrafish dataset, the difference is inverted compared to the human dataset: 46.69% overall, 38.13% in predicted nonsense regions, and 48.67% in remaining regions.

A new question arises whether the positive difference between prediction of nonsense and prediction of ID for human XP sequences is specific for XP sequences, or whether it can also be observed in synthetic nonsense sequences, or even in the false positive regions in NP sequences predicted to be nonsense. To answer this question, in each of the three groups of sequences we calculate the Pearson correlation coefficient *ρ *between predicted disorder content and predicted nonsense content for all sequences, and calculate *R*^2 ^statistic and *p*-value for linear regression. These indicators of correlation between prediction of nonsense and prediction of disorder for NP, XP and synthetic nonsense sequences are listed in Table 5. There is a significant positive correlation between predicted nonsense and predicted disorder in XP sequences in Homo sapiens. Surprisingly all correlation coefficients (including for XP sequences) in Danio rerio, as well as all correlation coefficients for NP sequences, are negative. However, the corresponding *R*^2 ^values are low.

**Table 5:** Correlation of disorder content (DC) and nonsense content (NC) for NP, XP and synthetic nonsense sequences

		NP			XP		*Synt. nonsense*		
Organism	**Corr. coeff**.	*R*^2^	*p*	**Corr. coeff**.	*R*^2^	*P*	**Corr. coeff**.	*R*^2^	*p*
Homo sapiens	-0.085	0.007	~0	0.354	0.125	~0	0.252	0.063	~0
Mus musculus	-0.123	0.015	~0	0.019	0.000	0.59	0.227	0.051	~0
Drosophila melanogaster	-0.120	0.014	~0	0.000	0.000	~0	0.098	0.010	~0
Danio rerio	-0.173	0.030	~0	-0.157	0.025	~0	-0.203	0.041	~0

Table contains Pearson correlation coefficients, and the *R*^2 ^statistics and *p*-values for linear regression of disorder content and nonsense content.

**Figure 8:**
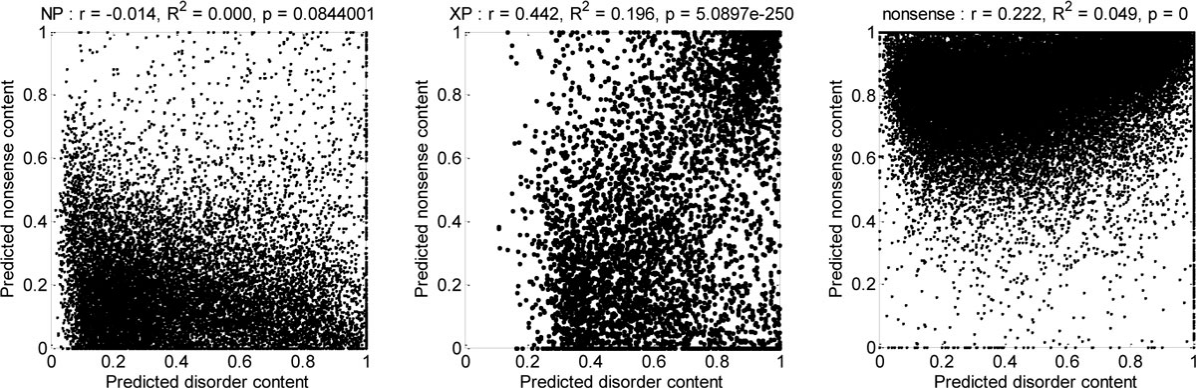
**Scatter-plots for predicted disorder content (x axis) vs. predicted nonsense content (y axis) for NP, XP, and synthetic nonsense sequences**. Results for the human dataset from the initial study [22] are shown. Pearson-correlation coefficients *r*, and *R*^2 ^statistics and p-values for linear regression models are shown above the plots.

It is interesting to note here that the correlation indicators for human XP sequences were much stronger in the initial study [22] (*ρ*=.442, *R^2^*=.196, and *p*~5E-250); therefore, we also produced scatterplots of predicted nonsense content against predicted disorder content (Figure 8). While the points representing NP sequences were clustered at the bottom (low level of prediction for nonsense) and the points representing synthetic nonsense sequences were clustered at the top (high level of prediction for nonsense), the points representing XP sequences form two clusters - in the upper-right corner (high disorder prediction, high nonsense prediction) and the lower right corner (low disorder prediction, low nonsense prediction). The decreased strength of the correlation may be attributed to the improved curation of the XP part of the human RefSeq sequences.

### Analysis of impact of input features for nonsense prediction

**Figure 9:**
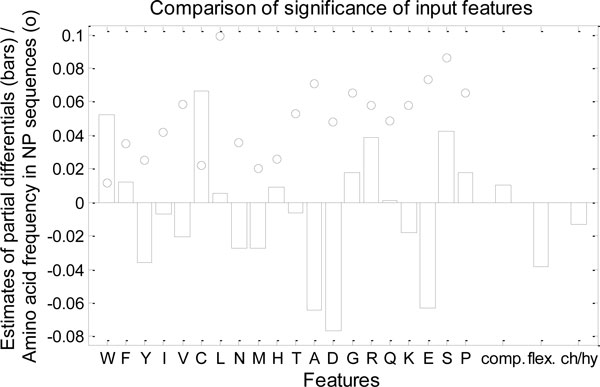
**Comparison of impact of input features**. Bars represent 23 approximated means of partial derivatives (with respect to 23 input features) over all points in the dataset. Circles represent the frequencies of the amino acids that the first 20 features are based on. The amino acids are ordered from most order promoting (left) to most disorder promoting (right).

The approximate means of partial derivatives of prediction function with respect to 23 input features over all points in the balanced training human dataset are shown in Figure 9. This figure also shows the frequencies of the amino acids corresponding to the first 20 input features, which are sorted according to their order (left) or disorder (right) promoting tendency. There is no obvious link between the sign and/or direction of the mean partial derivatives on one side and the amino acid frequencies and/or their order-disorder promoting property. Both positive and negative values are present among both the disorder promoting and order promoting amino acids. There are several pairs of amino acids with very similar frequencies and very different values of mean partial derivatives.

## Discussion

In a previous [16] we have observed a big increase in predicted disorder content for human protein sequences from NCBI with XP identifiers, as compared to human protein sequences with NP identifiers (Figure 2). This difference was consistent with the divergence in amino acid composition for NP and XP sequences (Figure 3), since several order-promoting amino acids were highly enriched in NP sequences, and several disorder-promoting amino acids were highly enriched in XP sequences.

Sequences have XP identifiers when they are in early stages of curation, and many of them are just putative sequences submitted by the automated genome annotation procedure that utilizes gene finding algorithms. Since gene finding algorithms are not perfect, they introduce nonsense regions into XP sequences. We suspected that these nonsense regions may be one of the causes for the discrepancy in predicted disorder.

Based on the difference in amino acid composition, we assumed that nonsense regions can be predicted from sequence. Since no data on nonsense regions was available, we developed a simple procedure to construct synthetic nonsense sequences from real protein sequences and their genomic sequences (Figure 4). These sequences have different amino acid composition than their real counterparts, although in human and mouse genome they also differ greatly from XP sequences, as they have higher fractions of some order-promoting amino acids and lower fractions of some disorder-promoting amino acids.

Using a simple prediction model, we have successfully trained predictors that discriminate true NP sequences from synthetic nonsense sequences. All input features were based only on local sequence information, and were constructed using methodology similar to many predictors of intrinsic disorder. The predictors have very good per-residue accuracies (82%-86%) and AUCs (> .9), comparable to predictors of intrinsic disorder (Table 2). More importantly, they are very well balanced (i.e. have similar sensitivity and specificity) and perform equally well on predicted disordered regions and predicted ordered regions, as well as on synthetic nonsense sequence regions originating from coding and non-coding genomic regions. These results confirm the assumption that nonsense regions can be predicted from sequence alone.

We have also used a simple method to aggregate per-residue predictions and obtain per-protein predictions. The performance of per-protein predictors is very close to optimal, with accuracies 96%-98% and AUC ~ .99. However, it is only feasible to use per-protein predictors when a sequence is either a true protein sequence or the whole sequence is nonsense.

We applied both per-residue and per-protein predictors to XP sequences. We used various methods to compare results of nonsense prediction for NP and XP sequences. Per-protein predictor classified ~44% of human XP sequences as fully nonsense sequences, compared to only ~6% of NP sequences. While this estimate is not realistic, it is indicative of how many XP sequences are - in terms of input features - more similar to synthetic nonsense sequences than to real NP sequences. Similar large discrepancy was observed for Mus musculus (~30% vs 7%), but not for Danio rerio (~9% vs 4%).

Per-residue predictor also gave very different predictions for human NP and XP sequences. The differences in distributions of nonsense content (fraction of residues in a sequence predicted to be in nonsense regions) are substantial for Homo sapiens and Mus musculus, but not for Danio rerio (Figure 7, Table 4).

We analyzed the total nonsense content (total fraction of residues in predicted nonsense regions) for NP, XP and synthetic sequences at various values of threshold. The separation margin between predicted nonsense contents for human NP and synthetic nonsense sequences peaks around the default threshold .5, and the margin between predicted nonsense contents for NP and XP is close to its maximum (~20% in mRNAnons, ~18% in GNMCnons dataset) at that threshold.

Predicted nonsense regions in human XP sequences have higher total disorder content (64.9%) than the remaining regions of human XP sequences (50.6%). More importantly, there is a significant positive linear dependency between predicted nonsense content and predicted disorder content in XP sequences, as indicated by fairly high Pearson correlation coefficient, as well as the *R*^2 ^statistic and low *p*-value for the corresponding linear regression model. While a similar positive linear dependency (albeit with lower correlation coefficient) is observed in synthetic nonsense sequences, it is completely absent from NP sequences. However, no such significant correlation can be observed in Mus musculus, while in Danio rerio the correlation is significant and negative. In Danio rerio, predicted nonsense regions in XP sequences have lower total disorder content (38.1%) than the remaining regions of human XP sequences (48.67%).

## Conclusions

The experimental results support the hypothesis that the presence of nonsense regions in human XP sequences - introduced by errors of gene finding procedures - significantly increases the predicted disorder content, and therefore introduces bias to genome-wide estimate of disorder content.

However, the same conclusion cannot be reached for Mus musculus and Danio rerio. Danio rerio has very similar distributions for predicted disorder content in NP and XP sequences, as well as very similar distributions for predicted nonsense content in NP and XP sequences. Furthermore, it has the lowest levels of predicted nonsense in XP sequences of all three compared organisms. Most importantly, the contribution of nonsense regions in XP sequences to predicted disorder content is at most minimal.

We were only able to partially explain the discrepancy in disorder content estimates for human NP and XP sequences. It is still possible that the proteins, which are currently covered with XP records, in fact have higher average disorder content than NP sequences. However, even if that is the case we cannot be sure what portion of the difference in predicted disorder content is due to the real difference, and what portion is due to errors in XP sequences that are to be eventually corrected. Differences in datasets and results for Homo sapiens, between the initial study [22] and the expanded study presented here, suggests that more and more XP sequences are being curated and eventually have they status upgraded, which leads to decrease in discrepancy between predicted disorder contents, as well as to lower predicted nonsense content.

## Competing interests

The authors declare that they have no competing interests.

## Authors' contributions

UM conceived, designed and carried out the study, and drafted the manuscript. ZO participated in the design of the study and helped to draft the manuscript.
